# Novel Arterivirus Associated with Outbreak of Fatal Encephalitis in European Hedgehogs, England, 2019

**DOI:** 10.3201/eid2702.201962

**Published:** 2021-02

**Authors:** Akbar Dastjerdi, Nadia Inglese, Tim Partridge, Siva Karuna, David J. Everest, Jean-Pierre Frossard, Mark P. Dagleish, Mark F. Stidworthy

**Affiliations:** Animal and Plant Health Agency–Weybridge, Addlestone, UK (A. Dastjerdi, N. Inglese, S. Karuna, D.J. Everest, J.P. Frossard);; Vale Wildlife Hospital and Rehabilitation Centre, Tewkesbury, UK (T. Partridge);; Moredun Research Institute, Penicuik, UK (M.P. Dagleish);; International Zoo Veterinary Group, Keighley, UK (M.F. Stidworthy)

**Keywords:** arterivirus, viruses, Arteriviridae, European hedgehog, Erinaceus europaeus, outbreak, meningitis/encephalitis, zoonoses, England

## Abstract

In the fall of 2019, a fatal encephalitis outbreak led to the deaths of >200 European hedgehogs (*Erinaceus europaeus)* in England. We used next-generation sequencing to identify a novel arterivirus with a genome coding sequence of only 43% similarity to existing GenBank arterivirus sequences.

Arteriviruses are enveloped, spherical viruses with a positive-sense, single-stranded, linear RNA genome (*1*), and they are assigned to the order Nidovirales, family *Arteriviridae*. Arteriviruses infect equids, pigs, possums, nonhuman primates, and rodents. For example, equine arteritis virus causes mild-to-severe respiratory disease, typically in foals, or abortion in pregnant mares (*2*). In pigs, porcine reproductive and respiratory syndrome virus types 1 and 2 cause a similar clinical syndrome of reproductive failure and respiratory disease (*3*,*4*). Wobbly possum disease virus causes an often fatal neurologic syndrome in possums (*5*). Several other arteriviruses (Pebjah virus, simian hemorrhagic encephalitis virus, and simian hemorrhagic fever virus) cause highly lethal hemorrhagic fever in captive Asian macaques (*6*). Lactate dehydrogenase–elevating virus was discovered by Riley et al. during their work on plasma enzyme levels in tumor-bearing mice (*7*). Arteriviruses were detected in Chinese softshell turtles (*Pelodiscus sinensis*) that had hemorrhagic disease (*8*) and from healthy African giant shrews (*Crocidura olivieri*) by molecular assays (*9*). Arteriviruses are documented to be transmitted through respiratory, venereal, and transplacental routes (*10*,*11*); direct contact with infected possums has been the most efficient route of wobbly possum disease virus transmission.

Arteriviruses were recently classified into 6 subfamilies (*Crocarterivirinae*, *Equarterivirinae*, *Heroarterivirinae*, *Simarterivirinae*, *Variarterivirinae*, and *Zealarterivirinae*) and 12 genera (*12*). The arterivirus genome is composed of a single, 12–16 kb, polyadenylated, RNA strand that contains 2 major genomic regions. The 5′ region contains open reading frames (ORFs) 1a and 1b coding for the viral polymerase and other nonstructural proteins (*13*). The 3′ region encodes the structural components of the virions and contains >7 ORFs. These ORFs code for the envelope protein, glycoproteins (2b–5), membrane, and nucleocapsid proteins. The 2 regions also differ in their protein expression mechanisms (*1*).

We describe the disease history, histopathology, and the near complete genome sequence of a novel arterivirus, hedgehog arterivirus 1 (HhAV-1). This virus was detected in association with fatal encephalitis in European hedgehogs (*Erinaceus europaeus*) from England.

## The Study

An outbreak of neurologic disease began in October 2019 in wild hedgehogs admitted to the Vale Wildlife Hospital and Rehabilitation Centre (Tewkesbury, England) and lasted for 4 months. These hedgehogs were from within a 50-km radius of the hospital. Those initially admitted were housed in a room dedicated to sick and young animals, sharing airspace with birds, rabbits, and occasionally rodents. Those admitted later were housed separately with other hedgehogs and occasionally with rabbits. Approximately 50% of hedgehogs admitted showed development of clinical signs, died, or were euthanized. Both juveniles and adults (≈15% of hedgehog admissions) were affected by this neurologic disease. In many instances, the animals became inappetent a few days after admission, although others took up to 6 weeks to become symptomatic.

Neurologic signs developed within 3 days of the onset of inappetence and included tremors, twitching, hyperaesthesia, ataxia/paresis, falling to 1 side, and paddling legs when laterally recumbent. Later signs included seizures, but most animals were euthanized before this stage. All described clinical signs developed after admission to the hospital; thus, all cases were considered nosocomial. Strict hygiene, biosecurity, and reduced juvenile admissions eventually resulted in the cessation of contagion. The outbreak resulted in >200 deaths.

The attending veterinarian at the wildlife hospital performed gross postmortem examinations of 3 newly dead hedgehogs. No major macroscopic lesions were identified. Histologic lesions in formalin-fixed brain were similar for all 3 hedgehogs (identification nos. 19-2271–3) examined by a specialist veterinary pathologist and consistent with a common etiology. Multiple coronal and longitudinal brain sections showed moderate-to-severe multifocal gliosis of highest severity in forebrain and hindbrain. Small numbers of neutrophils intermingled with microglia in perivascular foci expanding into the surrounding parenchyma. Brainstem, cerebral cortex, hippocampus, and midbrain contained similar lesions. Minimal multifocal meningitis with mononuclear inflammatory cell cuffing (intermingled with small-to-moderate numbers of neutrophils) was also observed. Ventricles (particularly midbrain) had subependymal edema and were infiltrated by mixed mononuclear cells and fewer neutrophils. Reactive astrocytes with conspicuous nucleoli were present within areas of gliosis and inflammation. Neuronal necrosis was occasionally observed.

Epithelia in many proximal renal tubules had intracytoplasmic lipid vacuolation and occasional intracytoplasmic protein globules. Moderate numbers of glomerular capsules and distal tubules contained eosinophilic, proteinaceous fluid with infrequent interstitial and perivascular neutrophils. Splenic red pulp was packed with abundant extramedullary hematopoietic cells. Plentiful periarteriolar lymphoid populations frequently included central lymphocytes with pyknotic nuclei. Liver and heart were histologically unremarkable. No fungi, protozoa, or viral inclusion bodies were recognized. Clinical manifestations, histologic characteristics, and distribution of lesions were distinct from those of wobbly hedgehog syndrome (*14*).

We performed immunohistochemical analysis (*15*) of formalin-fixed brain tissue from the 3 hedgehogs for *Listeria* spp., louping-ill, and related flavivirus antigens. All results were negative.

Virologic investigation was conducted at the Animal and Plant Health Agency–Weybridge (Addlestone, UK). Freshly frozen brain tissues from 3 additional hedgehogs (identification nos. 3375, 4896, and 3777) initially were tested for herpesviruses and showed negative results. The 3 samples were then subjected to next-generation sequencing (NGS) by using an Illumina MiSeq (https://www.illumina.com). The HhAV-1 sequence was obtained by de novo assembly using the SeqMan NGen (DNASTAR, Inc., https://www.dnastar.com). No other microbial pathogen was detected.

**Figure 1:**
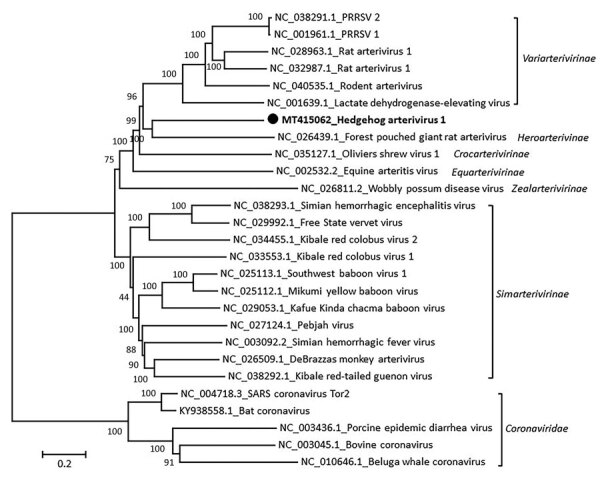
Phylogenetic analysis of the coding sequence of hedgehog arterivirus 1. The virus genome was aligned by using the MegAlign software of the DNASTAR Lasergene Core Suite (DNASTAR, Inc., https://www.dnastar.com), and phylogenetic analysis was performed by using MEGA 5.2 software (https://www.megasoftware.net). The rooted tree was constructed by using the neighbor-joining method and 1,000 bootstrap replications. Each virus on the tree is represented by its GenBank accession number and name. Designation of subfamilies was conducted as outlined in the International Committee on Taxonomy of Viruses 2018 release (https://talk.ictvonline.org/ictv-reports). Coronaviruses are included as an outgroup. Solid black circle and bold indicate strain detected in this study. Numbers along branches are bootstrap values. Scale bar indicates nucleotide substitutions per site.

**Figure 2:**
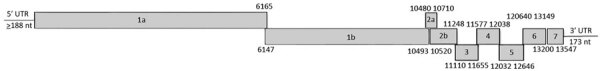
Genomic organization of hedgehog arterivirus-1. The genome arrangement resembles those of classical arteriviruses with open reading frames (ORFs) 1a/1b, 2a, 2b, 3, 4, 5, 6, and 7 that encode the polyproteins 1a/1ab, envelope protein, glycoproteins 2b–5, and membrane and nucleocapsid proteins, respectively. ORF1a and ORF1b are joined through a −1 ribosomal frameshift, encoding replicase precursor polyproteins pp1a/pp1ab. UTR, untranslated region.

The 3 identical HhAV-1 (UK 2019 strain) sequences had a genome coding sequence of >13,873 nt (GenBank accession no. MT415062). Genetic analysis of the sequence showed the highest similarity to arteriviruses detected in African giant-pouched rats (*Cricetomys gambianus)* (GenBank accession no. NC_026439) sampled in Guinea, but only 43% nt identity. Accordingly, the virus clustered phylogenetically with arterivirus in the African giant-pouched rat in the subfamily *Heroarterivirinae* (Figure 1). The virus genomic organization was determined to be typical of arteriviruses, in particular murine arteriviruses, and included ORF1a and 1b encoding replicase precursor polyproteins pp1a/pp1ab, followed by genes encoding envelope protein, the major structural glycoproteins (2b-5), matrix, and nucleocapsid proteins (Figure 2). At the amino acid level, the highest similarity was for the pp1b protein, where it showed a 50.6% similarity with the sequence from the African giant-pouched rat arterivirus.

To detect and quantify HhAV-1 load in samples from animals, we used reverse transcription quantitative PCR, primers forward 5′-CAG GAA CCC TCA CAG TAG-3′ and reverse 5′-TAA GAA GTT TGY GGC ATA G-3′, and probe (fluorescein) 5′-GGT TTC GTT CAA TGT TGA GGT-3′ (MGBEQ), which amplified a 100-nt segment of the ORF7 gene. Blood, brain, liver, lung, and spleen from these 3 animals were also positive for HhAV-1 in this PCR. The tissues tested had relatively high viral loads, and blood and brain had the highest load (measured by using the cycle threshold:β-actin ratio) but not much higher than those for other tissues.

## Conclusions

We detected a novel pathogenic arterivirus in the ever expanding family *Arteriviridae*. Because no wild animals were identified as having neurologic signs, we infer that all cases were probably hospital acquired. Whether the virus was introduced to the hospital through an asymptomatic carrier hedgehog or other wildlife (e.g., birds, rabbits or rodents) that shared the same airspace in the hospital remains unknown. However, arteriviruses are known to cause persistent/asymptomatic infections (e.g., equine arteritis virus, simian hemorrhagic fever virus, lactate dehydrogenase–elevating virus) and to be highly species specific (*10*,*13*). Therefore, the virus was most likely introduced by 1 or several asymptomatic hedgehogs.

This disease outbreak with neurologic signs highlights the requirement for strict biosecurity measures during rehabilitation involving intensive hospitalization of animals of this species, which are a frequent wildlife casualty submission in the United Kingdom. Hedgehogs are protected by the Biodiversity Action Plan in the United Kingdom.
